# The Oral Hygiene Problem

**Published:** 1912-03-15

**Authors:** Geo. E. Hunt


### THE ORAL HYGIENE PROBLEM.

BY GEO. E. HUNT, M.D., D.D.S.

What is the best way to push the propaganda before the people and thus hasten the time when its benefits will be accruing to the people of the nation? You know the truth. I know the truth, and a few hundreds of enlightened physicians and sociologic workers know the truth. But how can we impress it upon the great mass of the people so that those occupying the "seats of the mighty," who can make or mar the early success of the movement, may be constrained to make and not to mar. A year ago, I wrote an article for the magazine I have the pleasure of editing, asking that same question, What is the best way? Before writing that article I had formulated in my own mind what would be the best and the quickest way, and I hoped that others, also, might see it as I did, but as they did not do so I begin to wonder whether my deductions and conclusions were at fault. Briefly, my opinion then, was about as follows:

No big movement for the amelioration of human ills of whatever character is ever brought about spontaneously, or even quickly. All such forward movements are the result of patience, persistence and infinite minute forward steps. I feared that many interested in the oral hygiene movement, seeing the great truths it compassed, were sanguine that mere statement of these seemingly self-evident facts, would be sufficient to secure their world-wide adoption as a creed. But the history of intellectual advancement is all opposed to such a hasty adoption of any ameliorative measure. To illustrate, there are people yet in these United States and in this twentieth century who oppose vaccination and serum therapy. So if it was true that great movements inevitably move slowly, we could only fully impress the masses of the people by a generation or two of persistent, consistant effort. I believe today, that that is the ultimate solution of the problem of having the matter of better hygienic oral conditions accepted by the general public, as I shall explain later. But in the meanwhile, is there a way to force better mouth conditions on the public? In my opinion, but one.

The first step in the early solution of our problem is to prove to the medical profession at large, that the dentist and the physician not only should walk hand in hand but must walk hand in hand. The physician needs the dentist even more than the dentist needs the physician. There are only very occasional cases where the dentist requires the aid of the physician to round out the perfectness of his treatment, but there are few days indeed that the physician in general practice, and even most specialists, would not be materially aided in their work of physically benefiting humanity, by seeking the co-operation of the dentist. The time has come when the obstetritian, the rhinologist, the otologist, the aurist, the genito-urinary specialist, even in specific cases, the specialist in gastric and intestinal disorders, and the general practitioner, will be remiss in their life work, if they ignore oral conditions. One of the best diagnosticians in the medical profession in Indiana said to me less than a month ago that he thought he was referring about 75 per cent of his cases to their dentist. Since this is so, one of our first cares should be to convince more of the medical profession of this truth. In this effort, I am pleased to say, we will receive the able and efficient aid of the most enlightened men in the medical profession. Success in this effort will largely increase the interest of the people in better mouth conditions. People will do things, if in fear of sickness or the grave, that they will ignore if the incentive to do is but a fear of pain sometime in the future, or the possibilty of what they consider a slight esthetic marring of their person. So that I consider the timely cooperation of the medical profession, inevitable as it finally will be, a *sine qua non* to the early solution of our problem. Their interests and our interests are so inextricably interwoven, if the members of both professions can be brought to see it, that the greatest good for humanity can only be attained by concerted action. That this is coming to pass is true. That it is coming very slowly is to be deplored.

I am free to confess, however, that to my mind the successful educating of the adults of our people, even with the able assistance of our confreres of the medical profession, is too monumental a task for achievement in one generation. When we review the past and recall how slowly the people accept the obvious facts which lead to better health and how stubbornly they persist in habits of life and methods of living which science has repeatedly shown will lead to disease and death, we can hardly hope to create a revolution in oral conditions in one or two decades. I would not, for an instant, deprecate the importance of any efforts being made in that line, for I believe that every convert now is worth a score in the hereafter, but I am not optomistic regarding widespread results in the immediate future, and I therefore believe that in connection with our educational work among the physicians, our public press campaigns, our public platform work and our office educational work, that our greatest efforts should be directed toward the education of the children, the future citizens who shall dictate the future sociologic policy of the state.

I believe that consciously or sub-consciously, this has been the thought of most workers in the campaign. More effort has been directed toward amelioration of oral conditions amongst the children than in any other line of endeavor. And much of it, I am convinced, has been ill-advised and harmful to the ultimate success of the cause, rather than beneficial. Wholesale examination of the mouths of the school children, in the future, can have but a local moral effect. For that purpose it is of great value and should be performed. The fact that the school children in New York or Chicago show a certain percentage of defective mouths is but a matter of academic interest to the citizen of St. Louis or Indianapolis, but when he learns that the school children of his own city, in schools where his own children attend, show a large percentage of defective mouths, his interest is enlisted at once. An atrocious murder in our own community invariably arouses our interest many fold more than an even more atrocious one remotely committed. So that I believe inspection of the school children's mouths is worth the time and trouble it requires for the moral effect it has upon the community in which it takes place. But the time when it will teach us anything concerning oral conditions among the children has long since passed.

Where, in my opinion, the work among the children has been ill-advised and harmful to the cause, has been in those communities where free clinics for the children of the poor have been established, flourished for a time and then died of inanition. The average reformer, in the first blush of the newly found knowledge of a great wrong being perpetrated upon humanity, hungers for immediate action in the direction of a tangible effort to redress the wrong. His mind leaps from unaccustomed contemplation of the error to immediate redress of it, which is commendable and eloquent of his goodness of heart, but frequently equally eloquent of his lack of worldly wisdom, especially in reforming things. Free dental clinics, instituted, conducted and supported solely by members of the dental profession, have yet to prove their success. The fine enthusiasm which leads to their establishment seldom survives the tempering fires of time. The smouldering remains of their immature beginnings dot the map of many of the United States. It was a logical and a foregone conclusion. No free dental clinic, financed and conducted solely by dentists, has stood the test of even a few years. I do not blame the men who started these charities. If there was a fault committed, it was the fault of judgment, a fault of the head and not of the heart. They saw a great economic need and believed all that was necessary for its permanent correction was to call it to the attention of the people. Their purposes were high and their hearts and minds were filled with a pure philanthropic fire but it was inevitable that the fine enthusiasm responsible for the beginning, should have slowly dwindled under the chastening lash of time, of expense, and of non-appreciation, until it spent itself. It takes a stern mold of man to steadily neglect his livelihood and spend his modest income in the pursuit of an ideal, no matter how lofty it be.

Free dental clinics are necessary and they will come. But they are of right a state affair and the state should support them. In many communities, no doubt, they can be secured by the co-operation of the dentists with charitable organizations or with philanthropists, as has already been done in New York City, Rochester, Newark, Boston, and other communities. Where individuals or organizations can be persuaded to assist in the work to an appreciable degree, or where the dentists have the assurance of such assistance if they prove the worth of the movement, by all means should clinics be established without waiting for the state to act, for to my mind, the establishment and continuance of free clinics is one of the last steps in the problem. But it is the state's work, and until the state undertakes it, the free clinic will be on a precarious and an insecure footing. The state is the beneficiary in making these children better physically, therefore the state should shoulder both the responsibility and the expense. The greater efficiency of the individual in childhood saves money for the state in educational expense; the greater efficiency in adult life either saves the state the expense of maintaining a non-producer, or increases its revenues from one who is a more nearly perfect producer. You may call this socialism if you will. I will not be offended. Personally, I call it common sense. On this point William H. Allen says, "There never will come a time when the most direct means of promoting health, education and opportunity will not be through government."

If we grant the above line of argument to be logically correct, how shall we achieve the desired result? Again, I urge co-operation between the dentist and the physician. Only a few states have a law calling for medical inspection of the school children and in fewer yet does the law make inspection compulsory. The usual history of a medical inspection law is that after some years of local agitation on the part of a few "cranks," a permissive law will be granted the people by a complaisant Legislature. This means that the law making body authorizes either the school boards or the health boards to appoint medical inspectors, under certain restrictions, if they so choose. Later, after much more agitation, the state gets compulsory medical inspection, under which law all children are required to be inspected by appointed physicians. Now, in my opinion, all laws permitting or compelling medical inspection permit the appointive powers to appoint dental inspectors, if they wish to exert their powers to that extent. If they do so wish, and express that desire by action, the matter of dental inspectors is solved. If they do not so wish, the matter of dental inspection is delayed, until the people or the appointive power, usually a board of health, is further enlightened and educated. You will find numerous political problems entering into the question. Whether the board of health or the school board has the final decision in the matter, you will note, as I have had reason to do, that the fact that you have nothing to offer politically, constitutes a barrier to the appointment of dental inspectors. Eternal agitation and persistent publicity of conditions will generally correct this condition.

Even if you are unable to compass the desired end at the time, you can look forward to the next step, namely, a permissive dental inspection bill to be passed by the next Legislature. Success in that means that the school authorities or the board of health have permission to expend a certain amount of the funds gathered by the taxation of the people for this specific purpose. It is then up to the dental profession in their several communities in that state to convince the proper authorities that such an expenditure is not only legitimate but wise.

In my opinion, the school authorities will be more open to conviction in this connection than the boards of health and where new laws are sought, I would recommend that the school authorities be named as the appointive powers. However, if the physicians in the community can be brought to see the facts as I have set them forth, the health board is likely to follow the sentiment of the local medical organization. But it is a fact that today the educational authorities are more receptive to our plea than are the health boards. Politics is the answer.

After permissive dental inspection, compusory dental inspection follows as a matter of course and is the next forward step in the campaign. But that achieved, what next? Inspection of the mouths of the children is but a step, and a very inconsequential one, materially, in the solving of the problem. Compulsory care of the children's mouths is the real goal. I hold that no parent has any more right to send his child to a school, public or private, to mingle with other children, with its mouth in an unhygienic condition, than I have to go into a school room with a mass of pathogenic germs and scatter them broadcast. So the eventual and correct outcome of this movement must, and of right will be, the compulsory care of the mouths of all children by the state.

I do not mean by that, that all children shall be cared for at the expense of the state, for that would be unreasonable, but I do mean that the state should and will be as responsible for the proper care of the mouths of the children as it now is for the cultivation of their minds. Whether the child is a pupil of the state, in the public schools, or not will have no bearing on the question. The great economic problem will prevail—shall our children grow up to be full-powered producers, or partial or non-producers.

When we reach this state, our oral hygiene problem will be solved and solved correctly, but not before then. We cannot solve it for the present generation of adults for many reasons. In the first place it is impossible to interest the masses of the people to the extent that they will demand dental attention. They know nothing and care less about becoming better producers. Economic problems like ours do not affect individuals to an appreciable degree, or at least the individual thinks they do not, hence no great interest is taken in them. When you talk to the average individual in all earnestness about the future economic aspect of the question, he wonders what your personal pecuniary interest may be in the problem and expects you to wind up with a recommendation of some tooth paste or brush you are selling. Again, I would say, I am talking of the masses. Of the occasional converts we gain among the adults, I have nothing but praise to offer, and I believe, as I have before stated, that one recruit now is worth a score hereafter, but the ultimate solution of the problem, the golden age of better mouth conditions, better use of the mouth, and the final great good of this whole movement, depends on our education of the coming generations.

It is remarkable, at least to me who have looked into the mouths of a great many children, how similar mouth conditions are among the children of the wealthy or well-to-do and the children of the poorer classes at the age of six, when most children are entering the public schools. At that age, barring the inevitable deviations in all generalizations, children are wondrously uniform in their mouth conditions. When the time comes that the children of those who can afford it are given the necessary attention by the family dentist and the others are given equally careful attention by the dentists for the state, from this age on, this portion of the great problem of how best to increase the efficiency of our producers and decrease the number of our non-producers, will have been solved.

When we can take charge of the mouths of all children at five or six years of age, either in private practice or school clinic, and can carry them on to fourteen or fifteen with commendable mouth conditions, a knowledge of mouth hygiene, and better than all, a knowledge of what to eat, how much of it to eat, and how to eat it, the human race in this country will have taken a long stride toward perfect efficiency.

And that is my solution of the oral hygiene problem. I would do what circumstance will permit in the education of the present adults. I do not belittle in the slightest the work that is being done and the results that are being achieved. It is a glorious work and the results are worth all the effort we are putting forth. But I would warn those who are hyper-optimistic that the final outcome is not a matter of weeks or months, or even a few years; that the ultimate greatest good is a matter of decades and that many of us now in the harness will have passed to the great beyond where prophylaxis is a living reality rather than an iridescent dream, before this much to be desired condition becomes universal. However, that should not be a matter for discouragement to us for all great problems consume years of time and the lives of scores of advocates, in their solution.

Any worker looking for immediate extensive results and so mentally small that he cannot contribute his mite for fear the end will not be achieved in time for his participation in the enjoyment of contemplating it, is unworthy a great movement and should be allowed to withdraw, unregretted, whenever his faint heartedness prompts it. But for those who love a great cause for its very greatness; who love humanity for its follies and errors; who have a broad interest in the sociologic and economic game of the greatest good for the greatest number in its widest sense and most pronounced expression, the oral hygiene movement offers one of the most attractive fields for work that one can imagine. The care of the mouths of the children of our nation cannot help but appeal to their reason, their hearts, their sympathies, and their love for economic efficiency.—*Dental Review.*

